# Evaluations of the Effects of Extremely Low-Frequency Electromagnetic Fields on Growth and Antibiotic Susceptibility of *Escherichia coli* and *Pseudomonas aeruginosa*

**DOI:** 10.1155/2012/587293

**Published:** 2012-04-02

**Authors:** B. Segatore, D. Setacci, F. Bennato, R. Cardigno, G. Amicosante, R. Iorio

**Affiliations:** Department of Biomedical Sciences and Technologies, University of L'Aquila, 67100 L'Aquila, Italy

## Abstract

We aimed to investigate the effects of exposure to extremely low-frequency electromagnetic fields (2 mT; 50 Hz) on the growth rate and antibiotic sensitivity of *E. coli* ATCC 25922 and *P. aeruginosa* ATCC 27853. The electromagnetic field treatment significantly influenced the growth rate of both strains when incubated in the presence of subinhibitory concentrations of kanamycin (1 *μ*g/mL) and amikacin (0.5 *μ*g/mL), respectively. In particular, at 4, 6, and 8 h of incubation the number of cells was significantly decreased in bacteria exposed to electromagnetic field when compared with the control. Additionally, at 24 h of incubation, the percentage of cells increased (*P. aeruginosa*∼42%; *E. coli*∼5%) in treated groups with respect to control groups suggesting a progressive adaptive response. By contrast, no remarkable differences were found in the antibiotic susceptibility and on the growth rate of both bacteria comparing exposed groups with control groups.

## 1. Introduction

In the modern society, greater use of technologies leads to increasing exposure to extremely low-frequency (ELF) electromagnetic fields (EMFs) generated by structures and appliances such as power lines and ordinary devices used inside house and work places. As consequence, the effects of ELF-EMFs on the biological functions of living organisms represent an emerging area of interest with respect to environmental influences on human health. In latest years, several studies have been performed to verify direct effects exerted by ELF-EMF on cell functions. Although results have been somewhat controversial, a variety of cell responses have been observed involving proliferation and differentiation [1–10], gene expression [11–14], modulation of the membrane receptors functionality [15–20], apoptosis [21–23], alteration in ion homeostasis [1, 6, 13, 24–26], and free radicals generation [25, 27–30].

Bacteria have also been used in the studies with ELF-EMF [31–50]. In particular, it has been demonstrated that ELF-EMF can negatively [34, 37, 42, 45, 50] or positively [41, 42, 45, 47, 48] affect functional parameters (cell growth and viability) and bacteria antibiotic sensitivity depending on physical parameters of the electromagnetic field (frequency and magnetic flux density) applied, the time of the exposure, and/or the type of bacteria cells used. The possibility of a synergistic and/or antagonistic effect evoked by the combination of the appropriately patterned magnetic fields and specific antibiotics deserves special attention in *light* of the risk that antimicrobial *resistance* poses to public health. Bacteria are becoming increasingly resistant to almost all presently available antibiotics and this aspect is becoming a worldwide problem of highest significance [51, 52]. According to these considerations, the study of effects of ELF-EMF on bacteria is essential not only for investigation of environmental stress influences on biological systems, but also to explore the possibility of controlling the sensitivity of bacteria toward antibiotics in the environment or in clinical laboratories.

We have therefore attempted to investigate the possible influence of ELF-EMF on growth and antibiotic sensitivity of reference strains. To this end, we exposed *E. coli* ATCC 25922 and *P. aeruginosa* ATCC 27853 to an ELF-EMF with a sinusoidal waveform of 2 mT amplitude and frequency of 50 Hz, commonly produced in urban environments and in work places. These representative strains were chosen as examples of well-characterized Gram-negative bacteria, widely distributed in the environment and clinically relevant in nosocomial infections. Therefore, we evaluated the *in vitro* effect of ELF-EMF on the growth rate and antibiotic sensitivity of these strains. In particular, we examined the biological response of exposed cells to kanamycin and amikacin, well-known inhibitors of protein synthesis, incubating bacteria in the presence of subinhibitory concentration of these antibiotics.

## 2. Materials and Methods

### 2.1. Strains

 The international reference strains *Escherichia coli* ATCC 25922 and *Pseudomonas aeruginosa* ATCC 27853 were used for the experiments.

### 2.2. Antimicrobial Agents

Kanamycin, amikacin, ampicillin, and ceftazidime were purchased from Sigma Chemical (St. Louis, MO); the other study compounds (levofloxacin, cefazolin, ceftriaxone, and moxalactam) were obtained from the respective manufacturers.

### 2.3. Electromagnetic Field Exposure System

**Figure 1:**
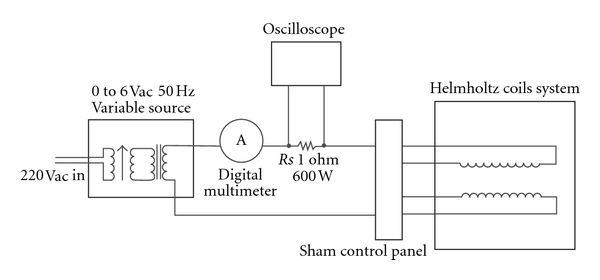
Experimental apparatus employed for oscillating magnetic field generation.

The exposure system consisted of an apparatus containing a pair of Helmholtz coils, a waveform generator, and a current amplifier (Figure 1). In our experiments, for the magnetic field generation we employed a pair of Helmholtz coils, with mean radius of 13.0 ± 0.5 cm. In each coil the number of turns was 800 with a 2 mm^2^ wire giving a resulting resistance of 2.4 Ω and an inductance of 39 ± 1 mH. The mean vertical distance between the coils was 13.5 ± 0.5 cm. The uniformity of the electromagnetic field was better than 1% within a cylindrical region that allowed a simultaneous exposure of a stack of four culture plates (Falcon multiwell plate; 96 wells) or twelve tubes of bacteria (20 mL glass tubes; effective sensitive volume ranging from 5 to 10 mL). This feature was in good agreement with the computation of the field distribution and homogeneity calculated by a Laplace equation simulation programme, which take into consideration the finite dimensions of coils. The generator was able to generate an effective magnetic field in the range 0–4 mT, with a sinusoidal wave of frequency of 50 Hz. The magnetic flux density (B) at the centre of coils was measured with an FW gaussmeter (Model 912, RFL Industries, Boonton, NJ) and B was adjusted by varying the coil current. The wave shape was visualized by an oscilloscope (Kikisui C0S5020) and the current flowing through the systems controlled by a digital multimeter (Agilent 34401A). The exposure system was put in an incubator at 37.0 ± 0.5°C. According the different connections, the current could either flow in the same direction or in the opposite direction (sham system), where the magnetic flux density is theoretically zero. In preliminary experiments (sham field experiments), we excluded any influence of the experimental device on environmental parameters such as temperature or gases tension. The frequency and flux density of the sinusoidal EMF were maintained at 50 Hz and 2.0 mT, respectively. To control the temperature, a thermometric sensor (Fluke 51-II, Fluke, WAQ3) was placed inside the Helmholtz coils system during the experiments measuring a constant temperature of  37.0 ± 0.3°C. Each sample, resuspended in the appropriate medium, was incubated in the presence (ELF-EMF exposed group) or absence (control group) of ELF-EMF. The ELF-EMF exposed group was placed in the core of the solenoid where a homogeneous sinusoidal magnetic field was generated, while control group was placed in a separate incubator.

### 2.4. Susceptibility Tests

Minimal Inhibitory Concentrations (MICs) were performed by conventional broth microdilution procedures in 0.1 mL volumes of Mueller Hinton broth. A final inoculum of 5 × 10^5^ colony-forming units (CFUs)/mL was used, as suggested by CLSI [53]. ELF-EMF exposed groups and control groups were incubated for 20 h at 37°C and then examined for cell growth. MIC results were recorded as the dilution value at which no visible growth occurred. As the growth curves were performed in glass tubes, MICs values were also determined by the broth macrodilution method (according to CLSI) using the same experimental parameters as those used for microdilution procedures. Data reported in Table 1 are referred to MIC values obtained using macrodilution procedures.

### 2.5. Growth Curves

The growth rates of *E. coli* and *P. aeruginosa* were carried out according to the method of Schoenknecht et al. [54]. ELF-EMF exposed groups and control groups were incubated in the presence or in the absence of subinhibitory concentration (1*⁄*4 × MIC) of 1 *μ*g/mL kanamycin (*E. coli*) and 0.5 *μ*g/mL amikacin (*P. aeruginosa*). Each sample (starting inoculum of about 5 × 10^5^CFU/mL opportunely diluted in 10 mL of Mueller Hinton broth) was incubated for 24 h. At 0, 4, 6, and 8 h of incubation samples were immediately vortexed for 15 sec and opportunely diluted. To test 24 h sample, at 20 h of incubation bacteria were vortexed for 15 sec and additionally reincubated for 4 h. At the end of incubation sample was immediately vortexed for 15 sec and opportunely diluted. After dilutions, one hundred microliters of each sample were plated and incubated for additionally 24 h at 37°C. At the end of the incubation the colony counts were performed and data were reported on semilog paper with the survivor colony count on the ordinate in logarithmic scale and the time in the abscissa in arithmetic scale.

### 2.6. Statistical Analysis

All experiments were replicated at least three times and the statistical significance of each difference observed among the mean values was determined by standard error analysis. The Sigma Stat 2.03 (SPSS, Chicago, IL) was used to test the statistical significance of differences between group means (one-way ANOVA followed by Tukey's test); *P* < 0.05 was considered to be statistically significant.

## 3. Results and Discussion

We tested *E. coli* and *P. aeruginosa* for their *in vitro *susceptibility to various antibiotics in the presence of ELF-EMF. On the basis of their different mechanism of action we evaluated the following classes of *antibiotics*: kanamycin, amikacin, ampicillin, cefazolin, ceftazidime, ceftriaxone, moxalactam, and levofloxacin.

**Figure 2:**
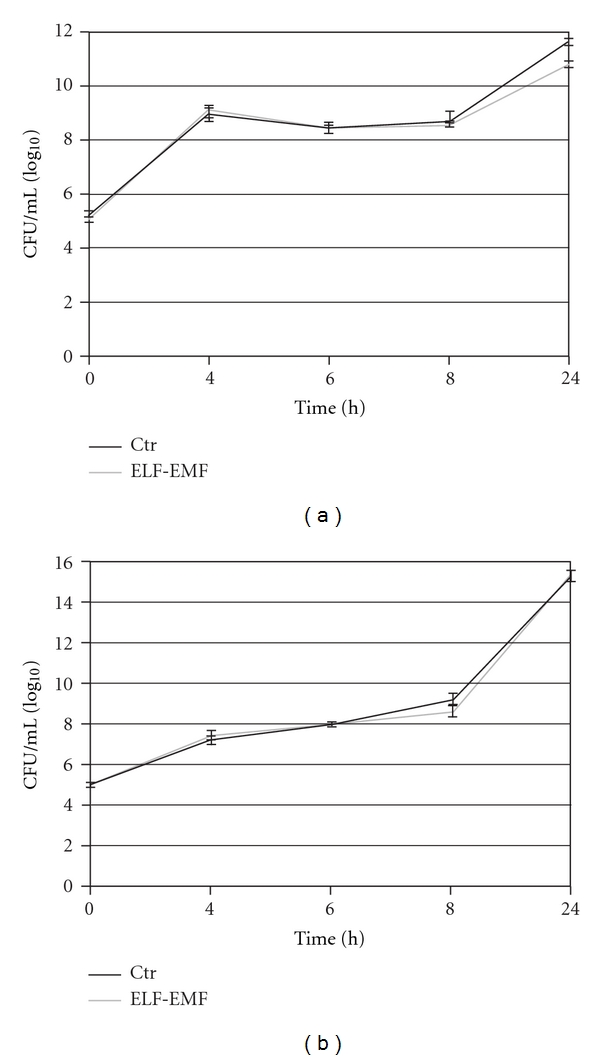
Effect of ELF-EMF (sinusoidal wave; 2 mT; 50 Hz) on growth rate of *E. coli *(a) and *P. aeruginosa* (b). ELF-EMF: ELF-EMF exposed groups; Ctr: control groups. Data represent means ± SEM from 3 different experiments.

Data obtained with untreated and treated cells did not reveal any significant changes in MIC values (Table 1) suggesting that under our experimental conditions long-term exposure (24 h) to ELF-EMF did not influence the degree of antibiotic susceptibility of *E. coli *and *P. aeruginosa*. We next examined the effect of ELF-EMF on the growth rate of bacteria. As shown in Figure 2, at each time point investigated, no remarkable differences were found in the rate of bacteria growth comparing exposed groups with control groups.

Our data do not support previous observations on the ability of ELF-EMF to induce changes of cell growth and antibiotic sensitivity that were reported for *E. coli* [37, 39, 42, 45–48, 55] and other strains [38, 39, 56]. In particular, it has been found that viability of different types of bacteria (*Escherichia coli*, *Leclercia adecarboxylata,* and *Staphylococcus aureus*) was affected after exposure to an ELF-EMF of 10 mT amplitude and frequency of 50 Hz [37]. Particularly, Gram-negative *E. coli* and *Leclercia adecarboxylata* achieved about 60–70% of colony forming units (CFU) number after exposure compared to the control ones. ELF-EMF (10 mT; 50 Hz) has lethal effect on bacteria *Paracoccus denitrificans*, but without changes in denitrification activity [39]. Additionally, Fojt and colleagues [38] have not observed any change in bacterial morphology neither of *E. coli* K12 (rod-like) nor of *P. denitrificans* CCM 982 (spherical) after exposure for 1 h to ELF-EMF (10 mT, 50 Hz) suggesting that bacteria shape does not play any important role in the interaction with magnetic field. On the contrary, it has been demonstrated that short-term exposure (20–120 min) to an ELF-EMF with a sinusoidal waveform of amplitude ranging from 0.1 to 1 mT and frequency of 50 Hz affected both cell viability and morphology of cultured *E. coli *ATCC 700926 [47]. In these experimental conditions, electromagnetic field also induced transcriptional changes and the acquisition of resistance to Cephalosporins (Cefuroxime and Ceftazidime). The influence of ELF-EMF on *E. coli* cultures was also studied by Justo and colleagues [42] which have found that cell growth could be altered (stimulated or inhibited) under magnetic field (100 mT; 50 Hz) effects. Further, the exposure of *E. coli* ATCC 25992 to an ELF-EMF of 2 mT amplitude and frequency of 50 Hz caused pronounced changes in the growth characteristic curves, morphology, structural properties of the extracted proteins, and the sensitivity and resistance to certain antibiotics such as amoxicillin, nalidixic acid, and erythromycin [45, 46]. These results were in agreement with the work of Stansell and colleagues [55] who found that moderate intensity static fields were able to cause a decrease in the antibiotic sensitivity and resistance of *E. coli* WHMC 4202. Additionally, Belyaev [48] showed that ELF-EMF, under specific conditions of exposure (frequency ranging from 8.5 Hz to 9 Hz; 0.021 mT), acted as a nontoxic but cell-growth stimulating agent on *E. coli* GE499. Again, the exposure of *E. coli* HB-101 to an ELF-EMF (25 mT; 6 Hz) produced a stimulation of cell growth [41]. By contrast, Grosman and colleagues [56] found that static magnetic fields ranging from 0.5 to 4.0 T had no significant influence on the growth rate and antibiotic sensitivity of *E. coli* and *Staphylococcus aureus*.

A direct comparison of these studies with our results is difficult because of the dissimilar experimental procedures employed. It is well known that the effects of ELF-EMF generally depend on both physical and biological parameters, including field signal characteristics (frequency, amplitude, wave shape, etc.), duration of exposure, cell metabolic state, genotype, and how long cells are allowed to grow before, during, and after exposure.

However, the apparent ineffectiveness to ELF-EMF exposure was, at least in part, confuted by the evaluations of the growth rate of bacteria in the presence or in the absence of subinhibitory concentration of antibiotics. This choice was not incidental* and based on* the hypothesis that the influence of ELF-EMF exposure could be bound to some soft modulation of the biological functions not detectable when bacteria were already exposed to excessive changes of stressful environments (MIC values of antibiotics). On the other hand, in absence of antibiotics bacteria may recognize electromagnetic stimulus and respond by activating a self-adjusting mechanism which allow them to maintain physiologically conditions. Thus, a possible cumulative effect could be detectable when bacterial cell was exposed to ELF-EMF and antibiotics at subinhibitory concentration all at once.

**Figure 3:**
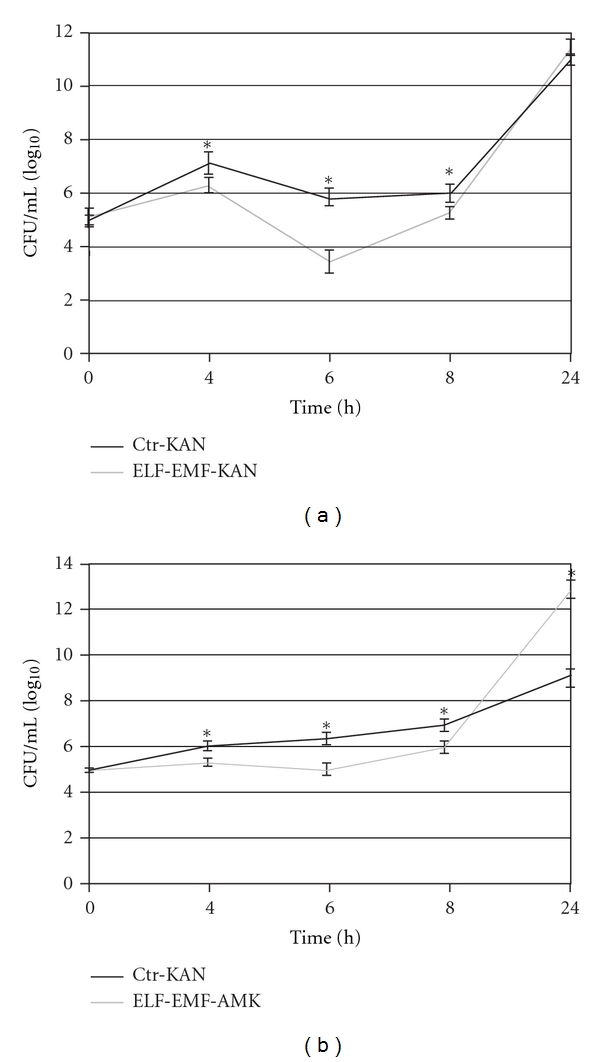
ELF-EMF (sinusoidal wave; 2 mT; 50 Hz) influenced the growth rate of *E. coli* (a) and *P. aeruginosa* (b) when incubated in the presence of subinhibitory concentration of kanamycin and amikacin, respectively. ELF-EMF-KAN: ELF-EMF exposed groups incubated in the presence of 1 *μ*g/mL kanamycin; Ctr-KAN: control groups incubated in the presence of 1 *μ*g/mL kanamycin; ELF-EMF-AMK: ELF-EMF exposed groups incubated in the presence of 0.5 *μ*g/mL amikacin; Ctr-AMK: control groups incubated in the presence of 0.5 *μ*g/mL amikacin. Data represent means ± SEM from 3 different experiments. *P < 0.05 (one-way ANOVA followed by Tukey's test).

Our data demonstrate that the exposure to ELF-EMF significantly influenced the growth rate of *E. coli* and *P. aeruginosa* when incubated in the presence of subinhibitory concentrations of kanamycin (1 *μ*g/mL) and amikacin (0.5 *μ*g/mL), respectively (Figure 3). In particular, at 4, 6, and 8 h of incubation the number of cells was significantly decreased in both strains exposed to ELF-EMF when compared with the control. The percentage of decrease for *E. coli* was 12 ± 2,  42 ± 5, and  13 ± 2  at 4, 6, and 8 h, respectively (Figure 3(a)). Similarly, the percentage of decrease for *P. aeruginosa* was 13 ± 2,  22 ± 3, and  14 ± 3  at 4, 6, and 8 h, respectively (Figure 3(b)). In both cases (*E. coli* and *P. aeruginosa*) ELF-EMF seemed to act in a similar way though some differences in the cell response were noted. In fact, at 6 h of incubation ELF-EMF exerted a more marked inhibitory effect on *E. coli* rather than *P. aeruginosa*. Moreover, at 24 h of incubation, the number of cells of *P. aeruginosa *significantly increased (~42%) in ELF-EMF exposed groups with respect to control groups, indicating a progressive and marked adaptive response to ELF-EMF. On the contrary, at 24 h of incubation, electromagnetic treatments tend to increase *slightly* the growth rate of *E. coli* (percentage of increase: ~5%) and the values were not significant.

From these data it resulted that ELF-EMF in combination of subinhibitory concentrations of antibiotics may act as stressing factor able to significantly affect the growth rate of bacteria. Moreover, to escape from these altered or stress-producing environments, bacteria can reverse (*P. aeruginosa*) or abolish (*E. coli*) their initial responses and seek to resume their normal level of homeostasis.

At this point different questions arise: (1) which cellular mechanism is responsible for coupling ELF-EMF to antibiotics in activating cell response?; (2) which cellular mechanism is involved in mediating the cellular adaptive response; (3) why does *P. aeruginosa* show a different response amplitude respect to *E. coli*? In this regard, it is possible to hypothesize an interaction between the electromagnetic field and the bacterial uptake process of aminoglycoside antibiotics. It is well known that aminoglycoside antibiotics are cationic molecules which binds to anionic components of the bacterial cell membrane in a reversible and concentration-dependent manner [57]. Therefore, the possibility that ELF-EMF could interfere with the surface charges of the membrane and/or the charge distribution on the antibiotic molecule modifying the rate of antibiotic penetration may exist, in view of the potential role played by ELF-EMF in modulating charge movements on the membrane. In this respect, it has been verified that ELF-EMF can affect membrane functions not only by a local effect on ion fluxes or ligand binding, but also by altering the distribution and/or the aggregation of the intramembrane protein [58–62]. However, we cannot exclude that ELF-EMF could interact with other specific processes that help the adaptation of bacteria to the new environment. In this regard, bacteria are able to respond to environmental stresses by activating suitable inducible systems, such as the DNA repair system, and exploit processes which increase the genetic variability.

Of note, since clinical and research laboratory instruments incorporate so many incontrollable electromagnetic fields, the observation that ELF-EMF did not affect the bacteria antibiotic sensitivity could exclude the possibility of producing alterations in laboratory test results where a high data reproducibility is required.

Further analyses are required to determine the molecular mechanisms underlying our early results. In this regard, it will be interesting to study the influence of different EMF frequency and/or intensity values on bacterial functional parameters to evaluate at which level the adaptive response starts. Moreover, in future studies, experiments involving strains with different genetic background will be investigated to elucidate our observations.

## Figures and Tables

**Table 1:** MIC values (*μ*g/mL) for *E. coli* and *P. aeruginosa* exposed or not exposed to ELF-EMF (sinusoidal wave; 2 mT; 50 Hz).

*Strains*				
	*E. coli*	*E. coli*/ELF-EMF	*P. aeruginosa*	*P. aeruginosa*/ELF-EMF
*Antibiotics*				
KAN	4	4	nd	nd
AMK	1	1	2	2
AMP	8	8	nd	nd
CFZ	4	4	nd	nd
CAZ	0.12	0.12	2	2
CRO	0.12	0.12	32	32
MOX	0.12	0.12	32	32
LVX	0.03	0.03	4	4

KAN: kanamycin; AMK: amikacin; AMP: ampicillin; CFZ: cefazolin; CAZ: ceftazidime; CRO: ceftriaxone; MOX: moxalactam; LVX: levofloxacin; *E. coli*: ATCC 25922; *P. aeruginosa*: ATCC 27853.
